# Floral Development Stage-Specific Transcriptomic Analysis Reveals the Formation Mechanism of Different Shapes of Ray Florets in Chrysanthemum

**DOI:** 10.3390/genes14030766

**Published:** 2023-03-21

**Authors:** Ya Pu, Minling Liao, Junzhuo Li, Yuankai Tian, Zhongman Wang, Xiang Song, Silan Dai

**Affiliations:** Beijing Key Laboratory of Ornamental Plants Germplasm Innovation & Molecular Breeding, National Engineering Research Center for Floriculture, Beijing Laboratory of Urban and Rural Ecological Environment, Key Laboratory of Genetics and Breeding in Forest Trees and Ornamental Plants of Education Ministry, School of Landscape Architecture, Beijing Forestry University, Beijing 100083, China

**Keywords:** ray floret, morphological difference, auxin-related genes, MADS-box, chrysanthemum, transcriptome analysis

## Abstract

The formation mechanism of different ray floret shapes of chrysanthemum (*Chrysanthemum* × *morifolium*) remains elusive due to its complex genetic background. *C. vestitum*, with the basic ray floret shapes of the flat, spoon, and tubular types, is considered a model material for studying ray floret morphogenesis. In this study, the flat and tubular type lines of *C. vestitum* at specific stages were used to investigate the key genes that regulate morphological differences in ray florets. We found that the expression levels of genes related to auxin synthesis, transport, and response were generally higher in the tubular type than in the flat type. *CvARF3* was highly expressed in the flat type, while *CvARF5* and *CvARF6* were highly expressed in the tubular type. Additionally, the transcription levels of Class B and E genes closely related to petal development, including *CvPI*, *CvAP3*, *Cvdefh21*, *CvSEP3*, and *CvCDM77*, were expressed at higher levels in the tubular type than the flat type. Based on the results, it is proposed that auxin plays a key role in the development of ray florets, and auxin-related genes, especially *CvARFs*, may be key genes to control the morphological difference of ray florets. Simultaneously, MADS-box genes are involved in the co-regulation of ray floret morphogenesis. The results provide novel insights into the molecular mechanism of different petal type formation and lay a theoretical foundation for the directional breeding of petal type in chrysanthemums.

## 1. Introduction

Chrysanthemum (*Chrysanthemum* × *morifolium*) has a wide variety of flower types and is a valuable ornamental and commercial crop. However, the genetic mechanism of chrysanthemum flower pattern formation remains elusive because of the complex genetic background. The most attractive part of the chrysanthemum is the unique capitulum composed of central disc florets and peripheral ray florets, whose morphology is susceptible to change due to internal and external factors [1]. The morphology and number of the ray florets principally determine the ornamental traits of the chrysanthemum [2]. The ray floret shapes are defined as the petal types, which are divided into flat, spoon, and tubular types according to the corolla tube merged degree (CTMD) [3,4]. Other complex petals, such as marginal elaboration and appendages of the corolla, evolved on the basis of these three basic petal types and were combined with their number, orientation, and location to form a rich variety of flower patterns. Therefore, the regulation mechanism of CTMD in ray florets could lay the foundation for the analysis of complex petal types and flower pattern formation. Petals experience four main processes during development: initiation, identity determination, morphogenesis, and maturation [5]. Plants construct different morphological petal structures by controlling the expression patterns of genes [6,7,8,9]. Therefore, elucidating the development and evolution of petals is the key to deciphering the diversification of petals and flowers [10]. The complex mechanism of single floral petals has been explored deeply, but the genetic mechanism of morphological variation of ray florets in chrysanthemums is still a mystery.

In order to find the key genes controlling ray floret types, a lot of forward genetics studies have been conducted. CTMD was an important morphological index for defining petal types [4] and could be described by a B-2 genetic model via two additive-dominance major genes [11]. Three major quantitative trait loci (QTLs) controlling CTMD were detected using a high-density genetic linkage map [12]. In genetic analysis of gerbera (*Gerbera hybrida*), it was found that the laciniated outer corolla lips of the ray floret were controlled by a dominant gene [13], while it was suggested that the CTMD in sunflower (*Helianthus annuus*) may be controlled by a pair of recessive genes [14]. Therefore, the genetic laws of the ray floret type in Asteraceae vary substantially due to the complexity of the genetic background and species differences.

In addition, studies on various ray floret types in Asteraceae have focused on the genetic regulation of petal symmetry, with the *CYCLOIDEA* (*CYC*) genes of the TCP family being research hotspots. Different ray floret types were closely related to the expression modifications of *CYC2s*. Studies on *Senecio vulgaris* [15], *H. annuus* [14,16,17], and *C. lavandulifolium* [18,19,20] have found that expression level changes or mutations of *CYC2s* could affect the ray floret types [16,21,22]. However, the function of *CYC2* genes in the ray florets of various Asteraceae plants was different, which could not completely explain the formation of different ray floret types.

According to numerous studies, ray floret morphology and flower patterns were influenced by plant hormones, such as auxin, ethylene, cytokinin (CTK), gibberellin (GA), abscisic acid (ABA), jasmonic acid (JA), and brassinosteroid (BR). Endogenous auxin derived the successive and centripetal initiation of floret primordia in an approximately circular pattern with a Fibonacci number, which influenced the number of florets [23]. Exogenous application of auxin led to homeotic conversions of florets and phyllaries [24]. The BR-related transcription factor BRI1-EMS-SUPPRESSOR 1 (BES1) had a repressive effect on organ boundary identity genes. In chrysanthemums, overexpressing *CmBES1* resulted in an increased fusion of the peripheral ray florets [25]. Additionally, JASMONATE ZIM DOMAIN (CmJAZ1), a JAZ repressor, could repress petal cell expansion and decrease the size of ray florets [26]. The above studies showed that plant hormones significantly regulate the morphology of ray florets.

In order to explore the key genes regulating the morphological differences of ray florets, *C. vestitum*, the closest hexaploid plant relative to chrysanthemum in this genus [27,28,29], was used for the transcriptome analysis. *C. vestitum* can be used as a model material to explore the formation mechanism of various ornamental characters of chrysanthemum [30,31], whose ray florets could be divided into the same basic types according to CTMD as chrysanthemum. In the previous study, stable *C. vestitum* lines of CVW with all ray florets being flat type and CVT with all ray florets being tubular type were obtained. Phenotypic observation revealed that stages 5–6 were the ray floret primordia formation period, stages 7–8 were the petal primordia development period, and stages 9–10 were the key stages for the formation of the difference between flat and tubular ray florets [30]. In order to explore the key genes regulating the different types of ray floret at early developmental stages, the stage-specific materials CVW and CVT were used for transcriptome sequencing. This study not only extends the knowledge of the regulation mechanisms of different ray floret types but also lays the theoretical foundation for directional breeding of flower types in chrysanthemums.

## 2. Materials and Methods

### 2.1. Plant Materials and Growth Conditions

**Figure 1:**
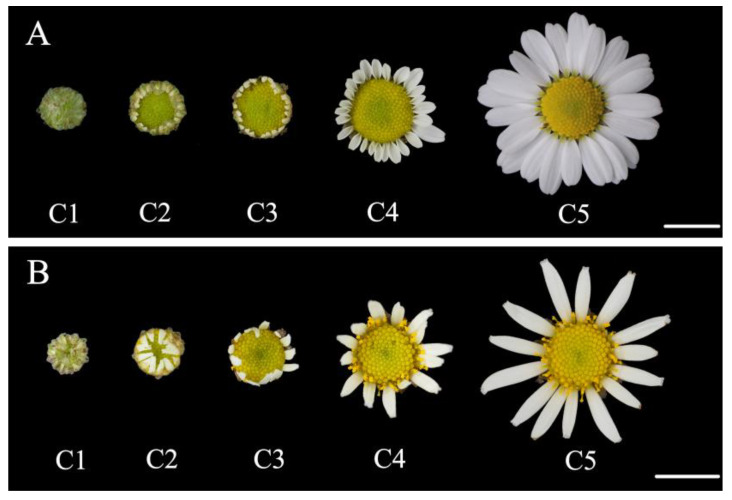
Characterization of flat-type line CVW and tubular type line CVT of *Chrysanthemum vestitum*. (**A**) The five different opening stages of the CVW capitula. (**B**) The five different opening stages of the CVT capitula. Scale bar = 1 cm.

The *C. vestitum* lines with different ray floret types distributed in the central China and were collected non-destructively through cuttings for ex-situ conservation [30]. The flat-type line CVW (CTMD = 0, Figure 1A) and the tubular-type line CVT (CTMD = 1, Figure 1B) of *C. vestitum* were grown in an artificial climatic chamber. During the vegetative growth period, plant materials were in long-day (LD) conditions (16 h light/8 h dark). And after completion of this period, plant materials were transferred to short-day (SD) conditions (12 h light/12 h dark). The temperature was maintained at 22 ± 1 °C, the air humidity was controlled at 40–50%, and the light intensity was 100–110 μmol·m^−2^·s^−1^.

### 2.2. RNA-Seq, Functional Annotation, and Data Processing

The materials were taken from the apical flower buds of CVW and CVT at specific stages, including stages 5–6 when the ray floret primordia were initiating and developing, stages 7–8 when the petal primordia of ray florets began to develop, and stages 9–10 when the morphological difference of ray florets was formed (Figure S1) [30]. A total of 18 libraries (W5-6, W7-8, W9-10, T5-6, T7-8, T9-10) were constructed for RNA-seq. In brief, total RNAs were extracted from the samples and cDNA libraries were sequenced on the Illumina NovaSeq 6000 sequencing platform (Illumina, San Diego, CA, USA) by Biomarker Technologies Corporation (Beijing, China). After connectors of the raw reads and low-quality sequences were removed, clean reads were obtained and then assembled using Trinity (version: v2.5.1, major parameter: --min_contig_length 200 --group_pairs_distance 500 --min_kmer_cov 1) [32]. The unigene sequences were aligned to the NR, Swiss-Prot, COG, KOG, eggNOG4.5, and KEGG databases using DIAMOND software (version: v2.0.4) [33] to obtain annotations. Raw sequence data were submitted to the National Center for Biotechnology Information (NCBI) Sequence Read Archive (SRA) database under accession number PRJNA934692.

### 2.3. Identification of Differentially Expressed Genes (DEGs)

The reads were compared with the unigene library using Bowtie2 (version: v2.3.5.1, major parameter: --no-mixed --no-discordant --gbar 1000 --end-to-end -k 200 -q -X 800) [34]; based on the results of the comparison, the expression levels were estimated using RSEM (version: v1.2.19) [35]. The transcript abundance of unigenes was estimated via the fragments per kilobase of transcript per million mapped reads (FPKM) [36]. DEGs analysis was performed by DESeq2 (version: v1.6.3, major parameter: default: test = “Wald”, fitType = “parametric”) [37] with the parameters that the false discovery rate (FDR) < 0.01 and the fold change (FC) ≥ 2.

### 2.4. Weighted Gene Co-Expression Correlation Network Analysis (WGCNA)

In order to screen genes involved in different ray floret types of *C. vestitum*, WGCNA analysis was performed with the procedure described by Lu et al. [38]. Subsequently, the modules with the highest correlation with the key stages of CVW and CVT were identified for further analysis. Genes in the module were subjected to KEGG analysis, and a hub gene co-expression network was constructed using Cytoscape software (version: v3.5.1) [39].

### 2.5. Real-Time Quantitative Polymerase Chain Reaction (qRT-PCR) Analysis

The expression patterns of DEGs were verified by the qRT-PCR. The differences in gene expression were verified in CVW and CVT capitula at different developmental stages (stages 5–6, 7–8, and 9–10). According to the SYBR Premix Ex Taq kit (Takara, Kyoto, Japan,), qRT-PCR analysis was performed on a CFX96™ real-time system (Bio-Rad Laboratories, Hercules, USA). Each qRT-PCR data point was derived from three technical replicates. The specific primer sequences for qRT-PCR were listed in Table S1. *CvSAND* was used as a reference gene to normalize the qRT-PCR data [40]. Relative expression levels were calculated using the 2^−ΔΔCT^ method [41], and the data are presented as the mean ± SD.

## 3. Results

### 3.1. Sequencing, Assembly, and Functional Annotation of the Transcriptome

**Figure 2:**
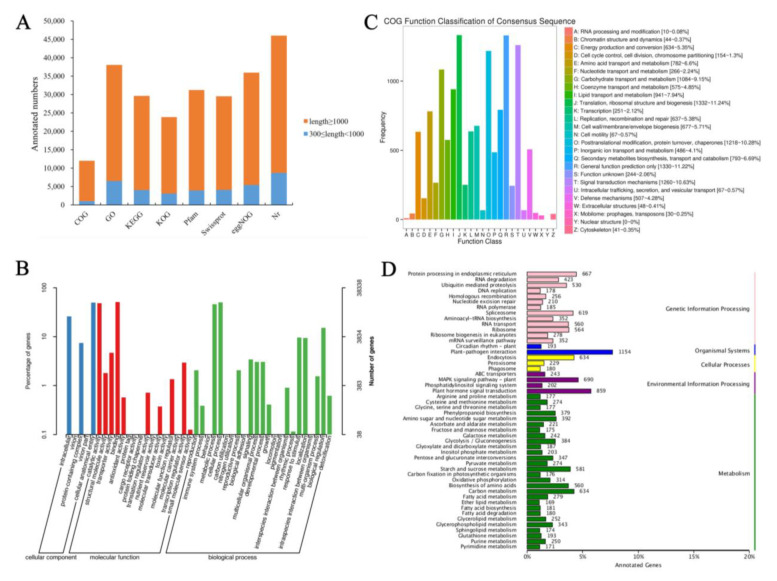
All genes are annotated in the public databases of CVW and CVT. (**A**) Statistics on the number of unigenes in different databases. (**B**) GO terms of unigenes. (**C**) COG function classification of unigenes. (**D**) KEGG pathways significantly enriched in unigenes.

To investigate the molecular mechanism of different ray floret types formation, stage-specific transcriptomes of CVW and CVT were carried out. The capitula of CVW and CVT at stages 5–6, 7–8, and 9–10 provided the templates for RNA-Seq analysis, and each group of samples contained three biological replicates. Summary statistics for 18 samples of sequencing data evaluations are shown in Table S2. A total of 125.09 Gb of clean data with a Q30 (percentage of sequences with sequencing error rates lower than 0.1%) of 94.88–95.78% was generated from 18 samples. Following assembly, 71,980 unigenes were obtained, of which 45,202 were above 1 kb in length, and the value of N50 was 2128 (Figure S2). A total of 47,348 unigenes were annotated, of which 23,980 (50.65%) were annotated in KOG, 31,299 (66.10%) were annotated in Pfam, 29,613 (62.54%) were annotated in Swissprot, and 46,385 (97.97%) were annotated in the Nr database (Figure 2A).

Functional classification of unigenes was performed using GO, COG, and KEGG assignments. 38,338 (80.97%) unigenes were divided into three functional groups (cell component, molecular function, and biological process) with a total of 44 categories by GO annotation (Figure 2B). The cellular component functional group had the most proteins associated with cellular anatomical entities and intracellular. A larger number of unigenes related to binding and catalytic activity were annotated in the molecular function group. The majority of proteins were related to cellular processes, metabolic processes, and biological regulation in the biological processes functional group. In addition, 12,071 (25.49%) unigenes were annotated in COG and classified into 25 functional groups (Figure 2C), among which the number of unigenes associated with translation, ribosomal structure and biogenesis, and signal transduction mechanisms were higher than those in other functional groups. A total of 29,780 (41.37%) unigenes were mapped into 136 KEGG pathways, with the most represented pathway being “plant-pathogen interaction (ko04626)”, followed by “plant hormone signal transduction (ko04075)” (Figure 2D).

### 3.2. Screening for the Genes Related to the Development of Different Ray Floret Types through WGCNA

**Figure 3:**
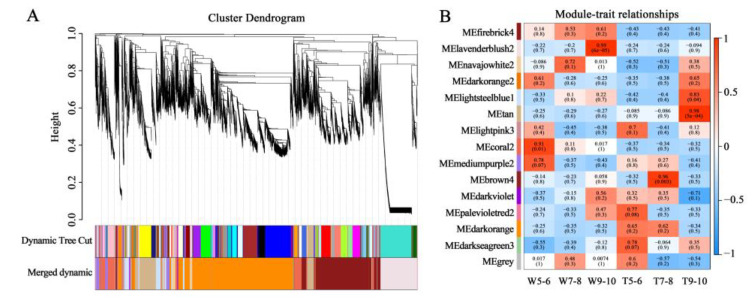
Weighted gene co-expression network analysis (WGCNA) of all unigenes in the transcriptome. (**A**) Dendrogram plot with color annotation. (**B**) Module-trait weight correlations and corresponding *p*-values. The color blocks on the left show different gene modules, and the color scale on the right shows module-trait correlation from low (blue) to high (red).

To understand the overall transcriptional profile during the development of ray florets with different CTMDs, WGCNA analysis was performed on the genes obtained by sequencing. According to the expression trends, the obtained genes were divided into 15 modules (Figure 3A), and the number of genes contained in each module is shown in Table S3. Eigengenes (the first major component in the module) represented the gene expression profile of the whole module, and 14 different expression patterns were obtained except for the gray module, the invalid module, with genes not involved in clustering (Figure S3). In previous studies, it was found that stages 9–10 are key stages for the formation of differences between flat and tubular ray florets. The results of the present study showed that the modules with the highest correlation with stages 9–10 of CVW and CVT were lavenderblush2 (correlation coefficient: 0.99) and tan (correlation coefficient: 0.98) (Figure 3B). It was speculated that the genes in these two modules may be closely related to the formation of ray floret morphological differences.

**Figure 4:**
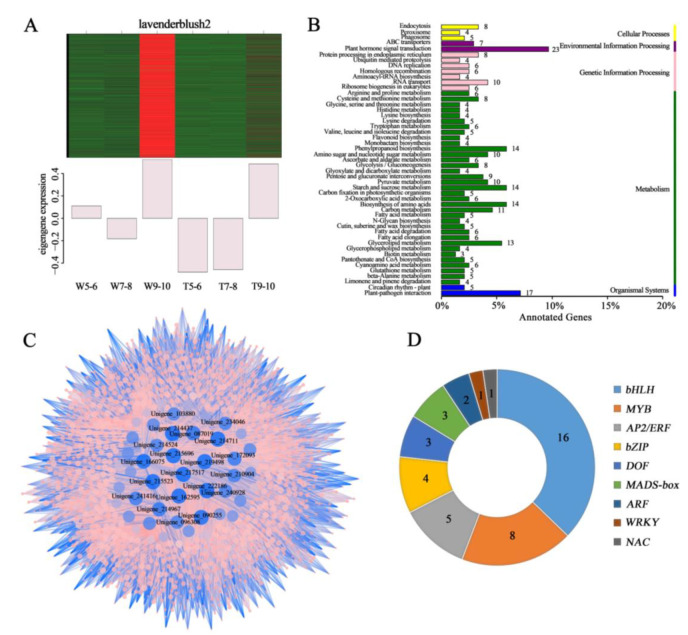
The expression pattern, annotation, and network prediction of genes in the lavenderblush2 module. (**A**) Expression pattern of eigengenes in the lavenderblush2 module. (**B**) KEGG pathways significantly enrich genes in the lavenderblush2 module. (**C**) Network of the top 50 hub genes from the lavenderblush2 module, which showed a high correlation with carotenoid accumulation. (**D**) Statistics on the number of important transcription factors in the lavenderblush2 module.

Further analysis of the lavenderblush2 module revealed that this module contained 2,281 genes and was abundantly expressed in stages 9–10 of the CVW (Figure 4A). A KEGG function analysis was performed on these genes. It was found that the most genes were enriched in the plant hormone signal transduction pathway (ko04075) (Figure 4B). The genes with KME values in the top 50 were selected to construct a co-expression network, and the hub genes in this network were screened according to their connectivity (Figure 4C). Furthermore, some transcription factors belonging to nine gene families were found, including basic helix-loop-helix (bHLH), MYBs, APETALA2/ETHYLENE RESPONSIVE FACTOR (AP2/ERF), basic (region-leucine) zipper (bZIP), DNA binding with one finger (DOF), MADS-box, AUXIN RESPONSE FACTOR (ARF), WRKY, and NAC (Figure 4D).

**Figure 5:**
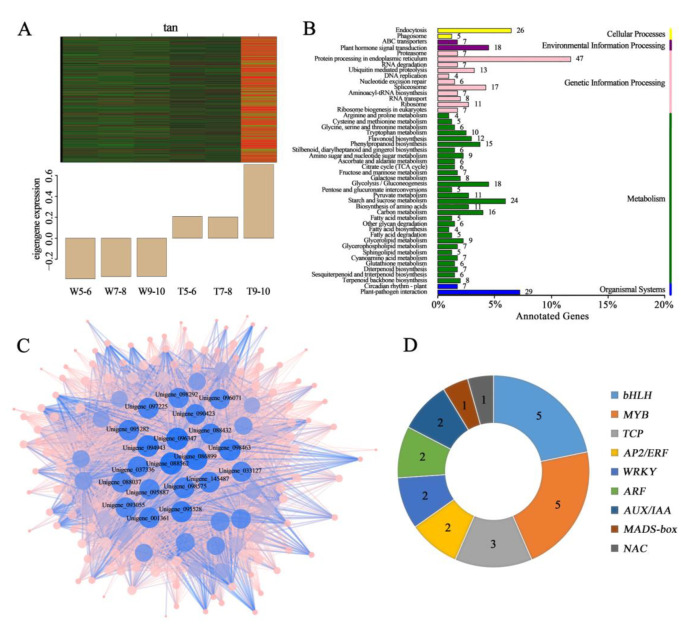
The expression pattern, annotation, and network prediction of genes in the tan module. (**A**) Expression pattern of eigengenes in the tan module. (**B**) KEGG pathways significantly enriching genes in the tan module. (**C**) Network of the top 50 hub genes from the tan module, which showed high correlation with carotenoid accumulation. (**D**) Statistics on the number of important transcription factors in the tan module.

The tan module contained 1602 genes, most of which were significantly expressed at stages 9–10 of the CVT (Figure 5A). KEGG analysis showed that these genes were enriched in the highest number of the protein processing pathways in the endoplasmic reticulum (ko04141) (Figure 5B). A co-expression network was constructed using the genes with KME values in the top 50, and the top 20 Hub genes with high connectivity to other genes were obtained (Figure 5C). Meanwhile, the statistics of transcription factors revealed that the tan module contained 5 bHLH, 5 MYB, 3 TCP, 2 AP2/ERF, 2 WRKY, 2 ARF, 2 AUXIN/INDOLE-3-ACETIC ACID (AUX/IAA), 1 MADS-box, and 1 NAC (Figure 5D).

### 3.3. DEGs Identified by K-Means Cluster Analysis

**Figure 6:**
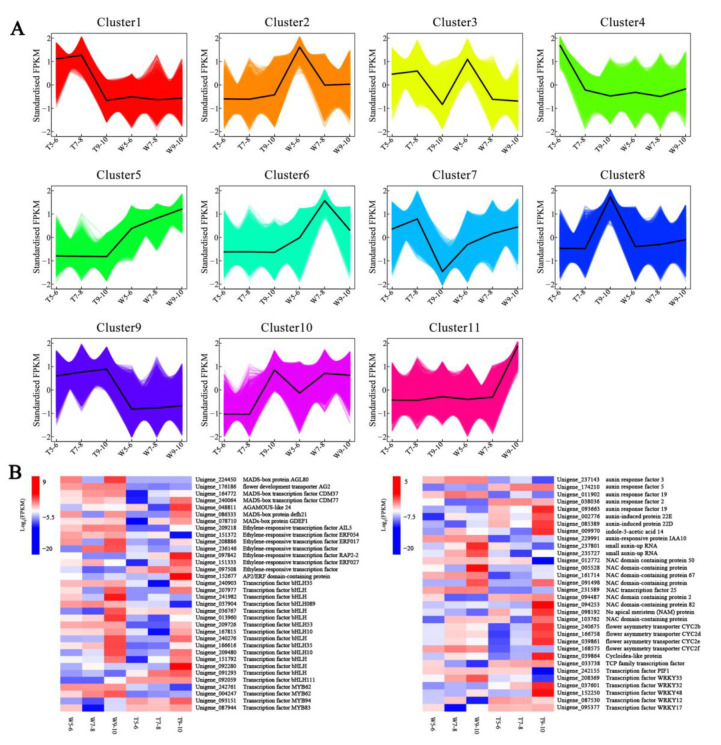
Cluster analysis of all differentially expressed genes (DEGs). (**A**) The eleven clusters of DEGs with different expression patterns. (**B**) Transcription factors detected by K-means cluster analysis.

The overall expression pattern of DEGs was shown on the clustering map with K-means cluster analysis, and all DEGs were classified into 11 clusters (Figure 6A). The genes in cluster 1 were expressed at high levels at stages 5–6 and 7–8 of CVT samples. The DEGs in clusters 2, 4, 8, and 11 showed high expression in W5-6, T5-6, T9-10, and W9-10 samples, respectively. The expression pattern of cluster 5 showed that there was no significant change in transcription level at different stages of CVT, while the expression level increased gradually in CVW with the continuous development of capitula. In addition, CVW and CVT had similar expression patterns in cluster 9, while the expression levels of CVT at different stages were significantly higher than those of CVW. Since the key stages of morphological differences in ray florets are stages 9–10 and genes involved in regulating ray floret development may function at earlier stages, it was suggested that the key candidate genes may be concentrated in clusters 5, 8, 9, and 11. Through K-means cluster analysis, 66 DEGs encoding transcription factors, including ARF, AP2/ERF, MADS-box, NAC, CYC2, bHLH, etc., were detected (Figure 6B).

### 3.4. Screening for Candidate DEGs Regulating Morphological Differences in Ray Florets by Pairwise Comparison

**Figure 7:**
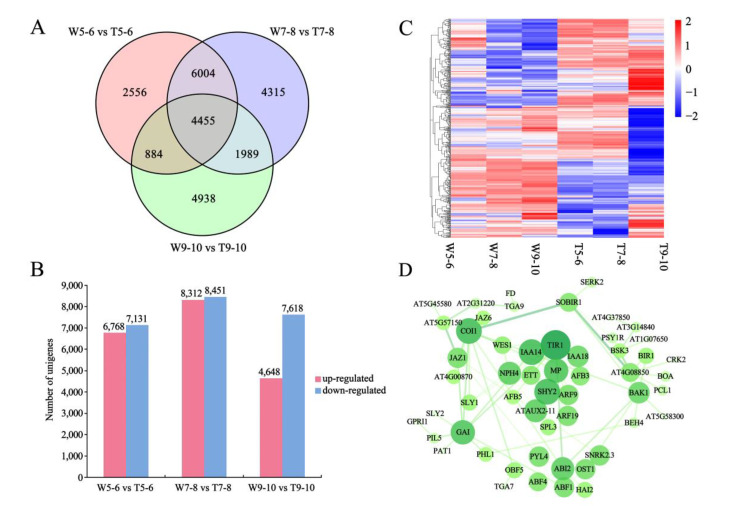
The differentially expressed genes (DEGs) of CVW and CVT at different developmental stages were identified by pairwise comparison. (**A**) A Venn diagram showing the number of DEGs revealed by paired comparison. (**B**) The number of up-regulated and down-regulated DEGs in comparisons among W5-6_vs._T5-6, W7-8_vs._T7-8, and W9-10_vs._T9-10. (**C**) A heatmap representation of the expression patterns of transcription factors. (**D**) Interaction network prediction of DEGs in plant hormone signal transduction (ko04075) pathway.

To identify more important genes regulating CTMD differences in ray florets, a pairwise comparison was performed between CVW and CVT. It was shown that there were a large number of DEGs between CVW and CVT at the same developmental stage in the Venn diagram (Figure 7A). A total of 25,141 DEGs were detected in the three comparisons, with 13,899 (6768 up-regulated and 7131 down-regulated), 16,763 (8312 up-regulated and 8451 down-regulated) and 12,266 (4648 up-regulated and 7618 down-regulated) DEGs in W5-6_vs._T5-6, W7-8_vs._T7-8, and W9-10_vs._T9-10, respectively (Figure 7B). Based on the developmental characteristics of different ray floret types, three gene sets are considered to be very important, including (i) genes differentially expressed at stages 5–6, 7–8, and 9–10; (ii) genes differentially expressed at stages 7–8 and 9–10; and (iii) genes differentially expressed at stages 9–10 only. The three gene sets contained 478 transcription factors, among which were 25 AP2/ERF family genes, 15 MADS-box family genes, 7 *NACs*, 6 *ARFs*, 5 AUX/IAA family genes, and 4 *TCPs* possibly involved in the regulation of morphological differences (Figure 7C). Furthermore, DEGs in the plant hormone signal transduction pathways of the three sets were screened, and STRING 11.5 (https://cn.string-db.org/, 6 June 2022) and Cytoscape software (version: v3.5.1) were used for interaction network analysis. It was found that DEGs were mainly auxin-related genes, including *TRANSPORT INHIBITOR RESPONSE* (*TIR1*, Unigene_033158), *IAA14* (Unigene_009970), *IAA3/SHORT HYPOCOTYL 2* (*IAA3/SHY2*, Unigene_085389), *MONOPTEROS/ARF5* (*MP/ARF5*, Unigene_174210), *ARF19* (Unigene_093663), *IAA18* (Unigene_034051), *ARF9* (Unigene_098607), and *ETTIN/ARF3* (*ETT/ARF3*, Unigene_237143) (Figure 7D). It was thus hypothesized that auxin may be the key factor affecting the morphology of ray florets.

Based on the above transcriptome analysis of CVW and CVT, some candidate DEGs were obtained in this study. The expression levels and annotations of these genes in different sequencing samples are shown in Table 1. Auxin related genes *ARF* and *AUX/IAA*, ethylene-related genes *AP2/ERF*, MADS-box family genes regulating flower development, *TCP* family genes regulating flower symmetry, and *NAC* family genes closely related to organ boundaries may be involved in the regulatory network of ray floret CTMD difference formation.

### 3.5. Validation of Key DEGs Related to Morphological Difference in Ray Florets

Based on the above results of the transcriptome analysis, it is hypothesized that the genes involved in plant hormone signal transduction, especially auxin-related genes, play an important role in regulating the development of different ray floret types. To determine the expression patterns of candidate genes in CVW and CVT, qRT-PCR analysis was performed in capitula at different developmental stages.

**Figure 8:**
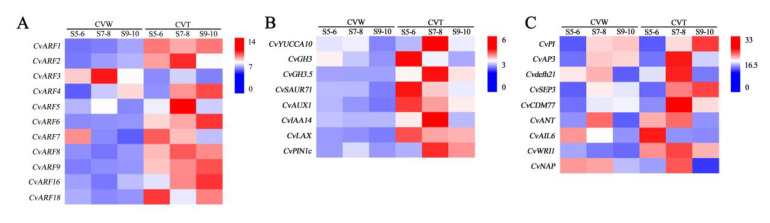
The expression patterns of DEGs in different samples were validated by real-time quantitative polymerase chain reaction (qRT-PCR). (**A**) Expression analysis of *CvARFs* in CVW and CVT at different developmental stages. (**B**) The expression patterns of genes related to auxin synthesis, transport, and response in CVW and CVT at different developmental stages. (**C**) Expression analysis of genes related to flower development in CVW and CVT at different developmental stages.

ARFs, as the core transcription factor connecting auxin signals and downstream genes, play a crucial role in the regulation of plant growth and development. Analysis of the expression pattern of *CvARFs* revealed that *CvARF1*, *CvARF6*, *CvARF8*, and *CvARF9* were all expressed at significantly higher levels in CVT at different developmental stages than CVW. And transcript levels of *CvARF2* and *CvARF5* were higher in CVT than CVW at stages 7–8, when petal primordia of ray florets were flourishing. However, the transcription level of *CvARF3* was higher in CVW, and this trend was also consistent with the results of a previous transcriptome (Figure 8A). CvARF5 and CvARF6, which belong to class II of the ARF family, usually activate downstream gene transcription in response to auxin, and CvARF3, which belongs to class I, usually functions as a transcriptional repressor. Their differential expression may be the cause of different ray floret types formation.

Moreover, the expression patterns of genes related to auxin synthesis, transport, and response were analyzed in this study. *CvYUCCA10* was expressed at the highest level in CVT at stages 7–8 and decreased at stages 9–10, similar to *GRETCHEN HAGEN 3.5* (*CvGH3.5*), *CvIAA14*, and *PINFORMED 1c* (*CvPIN1c*). *CvGH3*, *SMALL AUXIN UPREGULATED RNA 71* (*CvSAUR71*), *CvAUX1*, and *LAX PANICLE* (*CvLAX*) were highly expressed in CVT at stages 5–6, which was the ray floret primordia formation period, and these genes were generally expressed at higher levels in CVT than in CVW (Figure 8B).

Transcription factors closely related to petal development were also screened from the transcriptome, including *PISTILLATA* (*CvPI*), *APETALA3* (*CvAP3*), *DEFH21* (*Cvdefh21*), *SEPALLATA 3* (*CvSEP3*), and *CvCDM77* from the MADS-box family; *AINTEGUMENTA* (*CvANT*); *AINTEGUMENTA-LIKE 6* (*CvAIL6*); and *CvWRI1* from the AP2/ERF family; and *NAC-activated by AP3/PI* (*CvNAP*) from the NAC family. *CvPI* was expressed at the highest level in CVT at stages 9–10, while *CvAP3* was at the highest level in CVT at stages 7–8. The expression of *CvSEP3* and *CvCDM77* in CVT at stages 7–8 and 9–10 was higher than in CVW. And *CvWRI1* was expressed at high levels in CVT at all developmental stages compared with CVW (Figure 8C).

## 4. Discussion

### 4.1. Auxin and Auxin-Related Genes Are Involved in Regulating the Formation of Different Ray Floret Types

In this study, transcriptome sequencing and gene expression analysis revealed that a large number of genes related to auxin were differentially expressed in ray florets with different CTMDs. Additionally, we discovered that *CvSAUR*, *CvGH3*, and *CvGH3.5* were differentially expressed among the flat, spoon, and tubular of the *C. vestitum* line CVZ [30]. All these results demonstrated that auxin and auxin-related genes were involved in regulating different ray floret types. Auxin plays a crucial role in petal formation and is essential for plant growth and development [42,43]. Auxin activity is necessary for the beginning of petal primordia [44,45]. The number and shape of single floral petals can be dramatically impacted by mutations in the auxin synthesis, polar transport, and response genes [46,47]. The mutation of *YUCs* related to auxin synthesis can lead to serious defects in flower development [48]. And disruption of auxin polar transport in *pin1* and *pinoid* (*pid*) mutants can lead to anomalies in the number and positioning of organs or partial failure of floral organ initiation [49,50]. In addition, ARFs are the core transcription factors of auxin signal transduction and also affect the development process of petals. In *Mimulus lewisii*, the up-regulation of *MlARF3* and *MlARF4* resulted in unfused corollas [51]. Meanwhile, the study on the Asteraceae plant *Matricaria recutita* found that auxin affected the initiation of ray florets [24]. And it was found that *CvARF3*, *CvARF5*, *CvARF6*, and genes involved in auxin synthesis and transport were differently expressed between flat and tubular ray florets in this study. Therefore, we speculated that auxin may play an important role in ray floret morphogenesis, and the auxin-related DEGs screened in this study, such as *CvARF3*, *CvARF5*, and *CvARF6*, may be the key genes regulating morphological differences of ray florets.

### 4.2. Multiple Plant Hormones Are Involved in Regulating the Morphology of Ray Florets

In addition to auxin, several plant hormones are usually involved in regulating petal development. It has been reported that the *AUXIN-REGULATED GENE INVOLVED IN ORGAN GROWTH (ARGOS)* acts upstream of *ANT* [52,53], which is an AP2/ERF transcription factor member of a subfamily that is associated with regulation of petal cell proliferation [54,55]. In addition, *AIL6* is activated by *MP*/*ARF5* [56], which has a redundant function with *ANT* and regulates petal development [57]. In this study, the qRT-PCR analysis revealed that *CvARF5* was expressed at a higher level in CVT than in CVW, but *CvANT* and *CvAIL6* did not show any significant differences between the two types. While *CvWRI1*, which belongs to the same gene subfamily as *CvANT* and *CvAIL6*, has a higher transcript level in CVT than in CVW, implying that it may be involved in regulating the formation of different ray floret types.

Studies on Asteraceae plants have also found that several plant hormones, including ethylene, CTK, GA, ABA, BR, and JA, also affect the ray floret types [58,59,60,61]. GhWIP2 in Gerbera acts as a transcriptional repressor, suppressing cell expansion and reducing the length of ray florets by modulating crosstalk between GA, ABA, and auxin [60]. In addition, CmJAZ1, a repressor of the JA signaling pathway, and CmBES1, the BR-related transcription factor, were involved in regulating the morphology of ray florets [25,26]. It is evident that numerous plant hormone signals and related genes are involved in the regulation of ray floret morphogenesis. The auxin-related genes, such as *CvARF3*, *CvARF5*, and *CvARF6*, and the AP2/ERF family gene *CvWRI1* identified in this study, may mediate auxin and ethylene signals to jointly affect the morphological differences in ray florets.

### 4.3. MADS-box Genes Affect the Morphogenesis of Ray Florets

MADS-box genes are widely involved in the regulatory network of floral organ development, in which class A, B, and E genes synergistically regulate petal development [62,63]. In this study, the expression patterns of B-class genes *CvAP3*, *CvPI*, and *Cvdefh21* showed significant tissue specificity, with expression levels higher in CVT than in CVW, while transcript levels of E-class genes *CvSEP3* and *CvCDM77* were also significantly higher in CVT than in CVW. MADS-box genes control capitulum development in Asteraceae plants [64,65,66]. *SEPALLATA*-like genes *GRCDs* have been shown to contribute to meristem determinacy as well as flower type differentiation [67,68], and they also have functional redundancy in the regulation of organ identity [69]. Suppression of *GGLO1*, *GDEF1*, and *GDEF2* expression resulted in the disappearance or degeneration of gerbera trans florets and outer whorls of ray florets [70]. The roles of some MADS-box genes in Chrysanthemum have been analyzed in previous studies [71,72]. Transcriptome and expression analysis revealed that some MADS-box genes influence the development of the ray floret and disc floret [73,74], and MADS-box genes involved in networks interact with *CYC2* genes involved in networks to synergistically regulate floret differentiation [75,76]. The above studies showed that MADS-box genes were found to be extensively involved in the regulatory network of capitulum development. The B-class and E-class genes were differentially expressed in flat and tubular types in this study and could play important roles in regulating the formation of different ray floret types.

## 5. Conclusions

Based on floral development stage-specific transcriptome analysis combined with gene expression analysis, it was found that auxin-related genes, such as *CvARF3*, *CvARF5*, and *CvARF6*, and some transcription factors involved in floral development, such as AP2/ERF, MADS-box, CYC2, NAC, and bHLH, were differentially expressed in CVW and CVT. The results suggest that auxin plays a key role in ray floret development. Furthermore, *CvARFs* and homeotic genes involved in petal development work together to regulate ray floret differentiation. Future work will focus on the function and regulatory relationships of these genes. In conclusion, this study raises the prospects of auxin in regulating the morphological differences of ray florets and lays the foundation for further study of molecular mechanisms.

## Figures and Tables

**Table 1:** Candidate genes regulating the formation of different ray floret types.

#ID	FPKM of Each Sample						Annotation
	W5-6	W7-8	W9-10	T5-6	T7-8	T9-10	
ARF							
Unigene_093663	0.97	1.22	0.77	2.23	1.57	5.49	Auxin response factor 19
Unigene_149292	10.00	10.42	11.45	29.13	28.39	39.25	Auxin response factor
Unigene_237143	20.92	23.70	23.73	13.30	15.75	10.88	Auxin response factor 3
Unigene_174210	2.89	3.65	3.37	8.14	11.27	11.45	Auxin response factor 5
AUX/IAA							
Unigene_034051	4.45	2.25	1.22	9.58	10.39	3.79	Auxin-responsive protein
Unigene_085389	3.19	2.30	3.80	5.67	4.32	9.10	Auxin-induced protein 22D
Unigene_009970	0.90	2.88	1.75	1.44	0.68	7.70	Indole-3-acetic acid 14
AP2/ERF							
Unigene_168891	3.48	7.13	5.40	5.95	5.83	0.86	AP2-like ethylene-responsive transcription factor
Unigene_209218	2.23	5.12	4.72	0.18	0.18	1.09	AP2/ERF protein
Unigene_033078	0.17	0.11	0.06	1.72	2.00	0.66	AP2/ERF protein
Unigene_053843	48.23	3.91	0.33	29.64	21.68	2.11	AP2/ERF protein
Unigene_173391	22.79	0.36	0.23	5.68	4.68	1.60	Ethylene-responsive transcription factor 5
Unigene_237527	0.34	0.32	7.63	0.40	1.07	0.95	Ethylene-responsive transcription factor 14
MADS-box							
Unigene_086333	0.40	0.00	0.46	0.00	0.00	2.04	MADS-box protein defh21
Unigene_240064	7.67	43.22	131.43	105.67	180.61	348.32	MADS-box transcription factor CDM77
Unigene_164772	29.55	75.39	88.59	0.71	11.79	20.35	MADS-box transcription factor CDM37
Unigene_007650	6.53	5.91	3.62	6.83	6.39	7.54	flower development transporter AP3
Unigene_209803	0.06	1.60	2.96	6.58	7.36	13.96	GRCD5 protein/SEP3
Unigene_176186	7.27	8.76	8.90	1.39	1.24	1.54	Flower development transporter AG2
TCP							
Unigene_240675	1.55	2.38	2.02	0.60	1.28	6.74	Flower asymmetry transporter CYC2b
Unigene_166758	0.64	1.47	1.31	0.09	0.49	7.18	Flower asymmetry transporter CYC2d
Unigene_039861	2.04	2.82	2.26	0.96	1.95	6.72	Flower asymmetry transporter CYC2e
Unigene_168575	2.00	2.64	2.26	0.33	0.60	0.59	Flower asymmetry transporter CYC2f
NAC							
Unigene_154909	54.56	9.71	5.95	72.13	88.18	16.07	NAC1
Unigene_231589	3.87	4.57	4.49	1.56	1.54	1.95	NAC transcription factor 25
Unigene_096105	0.42	0.17	0.12	1.52	1.49	1.44	No apical meristem protein
Unigene_037350	2.39	0.13	0.08	4.47	5.33	13.66	No apical meristem protein

## Data Availability

The datasets supporting the results presented in this study are included in this article and its additional files.

## Supplementary Materials

The following supporting information can be downloaded at: https://www.mdpi.com/article/10.3390/genes14030766/s1. Figure S1: Schematic diagram of the capitulum development process of *Chrysanthemum vestitum* (Pu et al., 2020). The diagram was drawn according to the observation results of capitula obtained with paraffin sections and a scanning electron microscope. The ray floret primordia are initiating and developing at stages 5–6, the petal primordia of ray florets are developing at stages 7–8, and the morphological difference between a flat ray floret and a tubular ray floret has formed at stages 9–10. Figure S2: The length distribution of unigenes. The horizontal coordinate indicates the different length intervals of unigenes; the vertical coordinate indicates the number of unigenes in a certain length interval. Figure S3: Expression patterns of eigengenes in different modules. Expression patterns of 12 modules are shown in this figure, with lavenderblush2 and tan being shown in Figure 4A and Figure 5A, respectively. Table S1: Primer sequences used in qRT-PCR experiments. Table S2: Summary statistics of clean reads in the transcriptome. Table S3: The number of genes contained in each cluster in weighted gene co-expression network analysis (WGCNA).

## References

[1] Nasri F., Zakizadeh H., Vafaee Y., Mozafari A.A. (2021). In vitro mutagenesis of *Chrysanthemum morifolium* cultivars using ethylmethanesulphonate (EMS) and mutation assessment by ISSR and IRAP markers. Plant Cell Tissue Organ Cult..

[2] Reynolds J., Tampion J. (1983). Double Flowers: A Scientific Study.

[3] Dejong J., Drennan D.L. (1984). Genetic analysis in *Chrysanthemum morifolium*. II. Flower doubleness and ray floret corolla splitting. Euphytica.

[4] Song X.B., Gao K., Fan G.X., Zhao X.G., Liu Z.L., Dai S.L. (2018). Quantitative classification of the morphological traits of ray florets in large-flowered Chrysanthemum. Hortscience.

[5] Shan H.Y., Cheng J., Zhang R., Yao X., Kong H.Z. (2019). Developmental mechanisms involved in the diversification of flowers. Nat. Plants.

[6] Huang T., Irish V.F. (2015). Temporal control of plant organ growth by TCP transcription factors. Curr. Biol..

[7] Varaud E., Brioudes F., Szecsi J., Leroux J., Brown S., Perrot-Rechenmann C., Bendahmane M. (2011). AUXIN RESPONSE FACTOR8 regulates *Arabidopsis* petal growth by interacting with the bHLH transcription factor BIGPETALp. Plant Cell.

[8] Cavallini-Speisser Q., Morel P., Monniaux M. (2021). Petal cellular identities. Front. Plant Sci..

[9] Riglet L., Gatti S., Moyroud E. (2021). Sculpting the surface: Structural patterning of plant epidermis. iScience.

[10] Fu X.H., Shan H.Y., Yao X., Cheng J., Jiang Y.C., Yin X.F., Kong H.Z. (2022). Petal development and elaboration. J. Exp. Bot..

[11] Song X.B., Zhao X.G., Fan G.X., Gao K., Dai S.L., Zhang M.M., Ma C.F., Wu X.Y. (2018). Genetic analysis of the corolla tube merged degree and the relative number of ray florets in chrysanthemum (*Chrysanthemum* × *morifolium* Ramat.). Sci. Hortic..

[12] Song X.B., Xu Y.H., Gao K., Fan G.X., Zhang F., Deng C.Y., Dai S.L., Huang H., Xin H.G., Li Y.Y. (2020). High-density genetic map construction and identification of the locus controlling flower-type traits in Chrysanthemum (*Chrysanthemum* × *morifolium* Ramat.). Hortic. Res..

[13] Kloos W.E., George C.G., Sorge L.K. (2004). Inheritance of the flower types of *Gerbera hybrida*. J. Am. Soc. Hortic. Sci..

[14] Chapman M.A., Tang S., Draeger D., Nambeesan S., Shaffer H., Barb J.B., Knapp S.J., Burke J.M. (2012). Genetic analysis of floral symmetry in Van Gogh’s sunflowers reveals independent recruitment of *CYCLOIDEA* genes in the Asteraceae. PLoS Genet..

[15] Kim M., Cui M.L., Cubas P., Gillies A., Lee K., Chapman M.A., Abbott R.J., Coen E. (2008). Regulatory genes control a key morphological and ecological trait transferred between species. Science.

[16] Fambrini M., Salvini M., Pugliesi C. (2011). A transposon-mediate inactivation of a *CYCLOIDEA*-like gene originates polysymmetric and androgynous ray flowers in *Helianthus annuus*. Genetica.

[17] Fambrini M., Bellanca M., Costa Muñoz M.C., Usai G., Cavallini A., Pugliesi C. (2018). Ligulate inflorescence of *Helianthus* × *multiflorus*, cv. Soleil d’Or, correlates with a mis-regulation of a *CYCLOIDEA* gene characterised by insertion of a transposable element. Plant Biol..

[18] Huang D., Li X.W., Sun M., Zhang T.X., Pan H.T., Cheng T.R., Wang J., Zhang Q.X. (2016). Identification and characterization of *CYC*-like genes in regulation of ray floret development in *Chrysanthemum morifolium*. Front. Plant Sci..

[19] Chen J., Shen C.Z., Guo Y.P., Rao G.Y. (2018). Patterning the Asteraceae capitulum duplications and differential expression of the flower symmetry *CYC2*-like genes. Front. Plant Sci..

[20] Shen C.Z., Chen J., Zhang C.J., Rao G.Y., Guo Y.P. (2021). Dysfunction of *CYC2g* is responsible for the evolutionary shift from radiate to disciform flowerheads in the *Chrysanthemum* group (Asteraceae: Anthemideae). Plant J..

[21] Broholm S.K., TäHtiharju S., Laitinen R.A.E., Albert V.A., Teeri T.H., Elomaa P. (2008). A TCP domain transcription factor controls flower type specification along the radial axis of the *Gerbera* (Asteraceae) inflorescence. Proc. Natl. Acad. Sci. USA.

[22] Bello M.A., Pilar C., Álvarez I., Sanjuanbenito G., Fuertes-Aguilar J. (2017). Evolution and expression patterns of *CYC*/*TB1* genes in *Anacyclus*: Phylogenetic insights for floral symmetry genes in Asteraceae. Front. Plant Sci..

[23] Zhang T., Cieslak M., Owens A., Wang F., Broholm S.K., Teeri T.H., Elomaa P., Prusinkiewicz P. (2021). Phyllotactic patterning of gerbera flower heads. Proc. Natl. Acad. Sci. USA.

[24] Zoulias N., Duttke S.H.C., Garcês H., Spencer V., Kim M. (2019). The role of auxin in the pattern formation of the Asteraceae flower head (capitulum). Plant Physiol..

[25] Cheng P.P., Liu Y.N., Yang Y.M., Chen H., Cheng H., Hu Q., Zhang Z.X., Gao J.J., Zhang J.X., Ding L. (2020). *CmBES1* is a regulator of boundary formation in chrysanthemum ray florets. Hortic. Res..

[26] Guan Y.X., Ding L., Jiang J.F., Jia D.W., Li S., Jin L., Zhao W.Q., Zhang X., Song A.P., Chen S.M. (2022). The TIFY family protein CmJAZ1-like negatively regulates petal size via interaction with the bHLH transcription factor CmBPE2 in *Chrysanthemum morifolium*. Plant J..

[27] Dai S.L., Wang W.K., Li M.X., Xu Y.X. (2005). Phylogenetic relationship of *Dendranthema* (DC.) des moul. revealed by fluorescent *in situ* hybridization. J. Integr. Plant Biol..

[28] Chen J.Y., Wang C.Y., Zhao H.E., Zhou J. (2012). The Origin of Garden Chrysanthemum.

[29] Luo C., Chen D.L., Cheng X., Liu H., Li Y.H., Huang C.L. (2018). SSR Analysis of genetic relationship and classification in Chrysanthemum germplasm collection. Hortic. Plant J..

[30] Pu Y., Huang H., Wen X.H., Lu C.F., Zhang B.H., Gu X.Q., Qi S., Fan G.X., Wang W.K., Dai S.L. (2020). Comprehensive transcriptomic analysis provides new insights into the mechanism of ray floret morphogenesis in Chrysanthemum. BMC Genom..

[31] Luo J., Wang H., Chen S.J., Ren S.J., Fu H.S., Li R.R., Wang C.Y. (2021). CmNAC73 mediates the formation of green color in chrysanthemum flowers by directly activating the expression of chlorophyll biosynthesis genes *HEMA1* and *CRD1*. Genes.

[32] Grabherr M.G., Haas B.J., Yassour M., Levin J.Z., Thompson D.A., Amit I., Adiconis X., Fan L., Raychowdhury R., Zeng D.Q. (2011). Full length transcriptome assembly from RNA Seq data without a reference genome. Nat. Biotechnol..

[33] Buchfink B., Xie C., Huson D.H. (2015). Fast and sensitive protein alignment using DIAMOND. Nat. Methods.

[34] Langmead B., Trapnell C., Pop M., Salzberg S.L. (2009). Ultrafast and memory-efficient alignment of shor tDNA sequences to the human genome. Genome Biol..

[35] Li B., Dewer C.N. (2011). RSEM: Accurate transcript quantification from RNA Seq data with or without a reference genome. BMC Bioinform..

[36] Trapnell C., Williams B.A., Pertea G., Mortazavi A., Kwan G., van Baren M.J., Salzberg S.L., Wold B.J., Pachter L. (2010). Transcript assembly and quantification by RNA Seq reveals unannotated transcripts and isoform switching during cell differentiation. Nat. Biotechnol..

[37] Love M.I., Huber W., Anders S. (2014). Moderated estimation of fold change and dispersion for RNA-seq data with DESeq2. Genome Biol..

[38] Lu C.F., Pu Y., Liu Y.T., Li Y.J., Qu J.P., Huang H., Dai S.L. (2019). Comparative transcriptomics and weighted gene co-expression correlation network analysis (WGCNA) reveal potential regulation mechanism of carotenoid accumulation in *Chrysanthemum* × *morifolium*. Plant Physiol. Biochem..

[39] Shannon P., Markiel A., Ozier O., Baliga N.S., Wang J.T., Ramage D., Amin N., Schwikowski B., Ideker T. (2013). Cytoscape: A software environment for integrated models of biomolecular interaction networks. Genome Res..

[40] Qi S., Yang L.W., Wen X.H., Hong Y., Song X.B., Zhang M.M., Dai S.L. (2016). Reference gene selection for RT-qPCR analysis of flower development in *Chrysanthemum morifolium* and *Chrysanthemum lavandulifolium*. Front. Plant Sci..

[41] Livak K.J., Schmittgen T.D. (2001). Analysis of relative gene expression data using real-time quantitative PCR and the 2^−ΔΔCT^ method. Methods.

[42] Blakeslee J.J., Rossi T.S., Kriechbaumer V. (2019). Auxin biosynthesis: Spatial regulation and adaptation to stress. J. Exp. Bot..

[43] Báez R.R., Nemhauser J.L. (2021). Expansion and innovation in auxin signaling: Where do we grow from here?. Development.

[44] Chandler J.W., Jacobs B., Cole M., Comelli P., Werr W. (2011). *DORNRÖSCHEN-LIKE* expression marks *Arabidopsis* floral organ founder cells and precedes auxin response maxima. Plant Mol. Biol..

[45] Heisler M.G., Ohno C., Das P., Sieber P., Reddy G.V., Long J.A., Meyerowitz E.M. (2005). Patterns of auxin transport and gene expression during primordium development revealed by live imaging of the *Arabidopsis* inflorescence meristem. Curr. Biol..

[46] Cheng Y.F., Dai X.H., Zhao Y.D. (2006). Auxin biosynthesis by the *YUCCA* flavin monooxygenases controls the formation of floral organs and vascular tissues in *Arabidopsis*. Genes Dev..

[47] Pekker I., Alvarez J.P., Eshed Y. (2005). Auxin response factors mediate *Arabidopsis* organ asymmetry via modulation of *KANADI* activity. Plant Cell.

[48] Chen Q.G., Dai X.H., De-Paoli H., Cheng Y.F., Takebayashi Y., Kasahara H., Kamiya Y., Zhao Y.D. (2014). Auxin overproduction in shoots cannot rescue auxin deficiencies in Arabidopsis roots. Plant Cell Physiol..

[49] Bennett S.R.M., Alvarez J., Bossinger G., Smyth D.R. (1995). Morphogenesis in *PINOID* mutants of *Arabidopsis thaliana*. Plant J..

[50] Brewer P.B., Howles P.A., Dorian K., Griffith M.E., Ishida T., Kaplan-Levy R.N., Kilinc A., Smyth D.R. (2004). *PETAL LOSS*, a trihelix transcription factor gene, regulates perianth architecture in the *Arabidopsis* flower. Development.

[51] Ding B.Q., Xia R., Lin Q.S., Gurung V., Sagawa J.M., Stanley L.E., Strobel M., Diggle P.K., Meyers B.C., Yuan Y.W. (2020). Developmental genetics of corolla tube formation: Role of the tasiRNA-*ARF* pathway and a conceptual model. Plant Cell.

[52] Hu Y., Xie Q., Chua N.H. (2003). The Arabidopsis auxin-inducible gene *ARGOS* controls lateral organ size. Plant Cell.

[53] Mizukami Y., Fischer R.L. (2000). Plant organ size control: *AINTEGUMENTA* regulates growth and cell numbers during organogenesis. Proc. Natl. Acad. Sci. USA.

[54] Horstman A., Willemsen V., Boutilier K., Heidstra R. (2014). AINTEGUMENTA-LIKE proteins: Hubs in a plethora of networks. Trends Plant Sci..

[55] Krizek B.A. (2015). *AINTEGUMENTA-LIKE* genes have partly overlapping functions with *AINTEGUMENTA* but make distinct contributions to *Arabidopsis thaliana* flower development. J. Exp. Bot..

[56] Yamaguchi N., Wu M.F., Winter C.M., Berns M.C., Nole-Wilson S., Yamaguchi A., Coupland G., Krizek B.A., Wagner D. (2013). A molecular framework for auxin-mediated initiation of flower primordia. Dev. Cell.

[57] Krizek B.A. (2009). *AINTEGUMENTA* and *AINTEGUMENTA-LIKE6* act redundantly to regulate Arabidopsis floral growth and patterning. Plant Physiol..

[58] Li L.F., Zhang W.B., Zhang L.L., Li N., Peng J.Z., Wang Y.Q., Zhong C.M., Yang Y.P., Sun S.L., Liang S. (2015). Transcriptomic insights into antagonistic effects of gibberellin and abscisic acid on petal growth in *Gerbera hybrida*. Front. Plant Sci..

[59] Huang G., Han M.X., Yao W., Wang Y.Q. (2017). Transcriptome analysis reveals the regulation of brassinosteroids on petal growth in *Gerbera hybrida*. PeerJ.

[60] Ren G.P., Li L.F., Huang Y.H., Wang Y.Q., Zhang W.B., Zheng R.Y., Zhong C.M., Wang X.J. (2018). GhWIP2, a WIP zinc finger protein, suppresses cell expansion in *Gerbera hybrida* by mediating crosstalk between gibberellin, abscisic acid, and auxin. New Phytolpgist.

[61] Wang J.J., Wang H.B., Ding L., Song A.P., Shen F., Jiang J.F., Chen S.M., Chen F.D. (2017). Transcriptomic and hormone analyses reveal mechanisms underlying petal elongation in *Chrysanthemum morifolium* ‘Jinba’. Plant Mol. Biol..

[62] Alvarez-Buylla E.R., Benítez M., Corvera-Poiré A., Chaos Cador A., de Folter S., Gamboa de Buen A., Garay-Arroyo A., Garcia-Ponce B., Jaimes-Miranda F., Perez-Ruiz R. (2010). Flower development. The Arabidopsis Book.

[63] Theißen G., Melzer R., Florian R. (2016). MADS-domain transcription factors and the floral quartet model of flower development: Linking plant development and evolution. Development.

[64] Irish V. (2017). The ABC model of floral development. Curr. Biol..

[65] Laitinen R.A., Broholm S., Albert V.A., Teeri T.H., Elomaa P. (2006). Patterns of MADS-box gene expression mark flower-type development in *Gerbera hybrida* (Asteraceae). BMC Plant Biol..

[66] Yu D., Kotilainen M., Pöllänen E., Mehto M., Elomaa P., Helariutta Y., Albert V.A., Teeri T.H. (1999). Organ identity genes and modified patterns of flower development in *Gerbera hybrida* (Asteraceae). Plant J. Ceel Mol. Biol..

[67] Uimari A., Kotilainen M., Elomaa P., Yu D., Albert V.A., Teeri T.H. (2004). Integration of reproductive meristem fates by a *SEPALLATA-like* MADS-box gene. Proc. Natl. Acad. Sci. USA.

[68] Elomaa P., Zhao Y.F., Zhang T. (2018). Flower heads in Asteraceae-recruitment of conserved developmental regulators to control the flower-like inflorescence architecture. Hortic. Res..

[69] Zhang T., Zhao Y.F., Juntheikki I., Mouhu K., Broholm S.K., Rijpkema A.S., Kins L., Lan T.Y., Albert V.A., Teeri T.H. (2017). Dissecting functions of *SEPALLATA*-like MADS box genes in patterning of the pseudanthial inflorescence of *Gerbera hybrida*. New Phytol..

[70] Broholm S.K., Pöllänen E., Ruokolainen S., Tähtiharju S., Kotilainen M., Albert V.A., Elomaa P., Teeri T.H. (2010). Functional characterization of B class MADS-box transcription factors in *Gerbera hybrida*. J. Exp. Bot..

[71] Sun C.H., Wang J.H., Gu K.D., Zhang P., Zhang C.S., Hu D.G., Ma F.F. (2021). New insights into the role of MADS-box transcription factor gene *CmANR1* on root and shoot development in chrysanthemum (*Chrysanthemum morifolium*). BMC Plant Biol..

[72] Sasaki K., Yoshioka S., Aida R., Ohtsubo N. (2021). Production of petaloid phenotype in the reproductive organs of compound flowerheads by the co-suppression of class-C genes in hexaploid *Chrysanthemum morifolium*. Planta.

[73] Liu H., Jia Y., Chai Y.H., Wang S., Chen H.X., Zhou X.M., Huang C.L., Guo S., Chen D.L. (2022). Whole-transcriptome analysis of differentially expressed genes between ray and disc florets and identification of flowering regulatory genes in *Chrysanthemum morifolium*. Front. Plant Sci..

[74] Fan J.W., Huang J.L., Pu Y., Niu Y.J., Zhang M.M., Dai S.L., Huang H. (2022). Transcriptomic analysis reveals the formation mechanism of anemone-type flower in chrysanthemum. BMC Genom..

[75] Wen X.H., Qi S., Huang H., Wu X.Y., Zhang B.H., Fan G.X., Yang L.W., Hong Y., Dai S.L. (2019). The expression and interactions of ABCE-class and *CYC2-like* genes in the capitulum development of *Chrysanthemum lavandulifolium* and *C.* × *morifolium*. Plant Growth Regul..

[76] Ding L., Song A.P., Zhang X., Li S., Su J.S., Xia W.K., Zhao K.K., Zhao W.Q., Guan Y.X., Fang W.M. (2020). The core regulatory networks and hub genes regulating flower development in *Chrysanthemum morifolium*. Plant Mol. Biol..

