# Supplementary figures and images for: Floral Development Stage-Specific Transcriptomic Analysis Reveals the Formation Mechanism of Different Shapes of Ray Florets in Chrysanthemum
Source: Genes (Basel). 2023 Mar 21;14(3):766. doi: 10.3390/genes14030766 (PMC10048392; doi:10.3390/genes14030766)

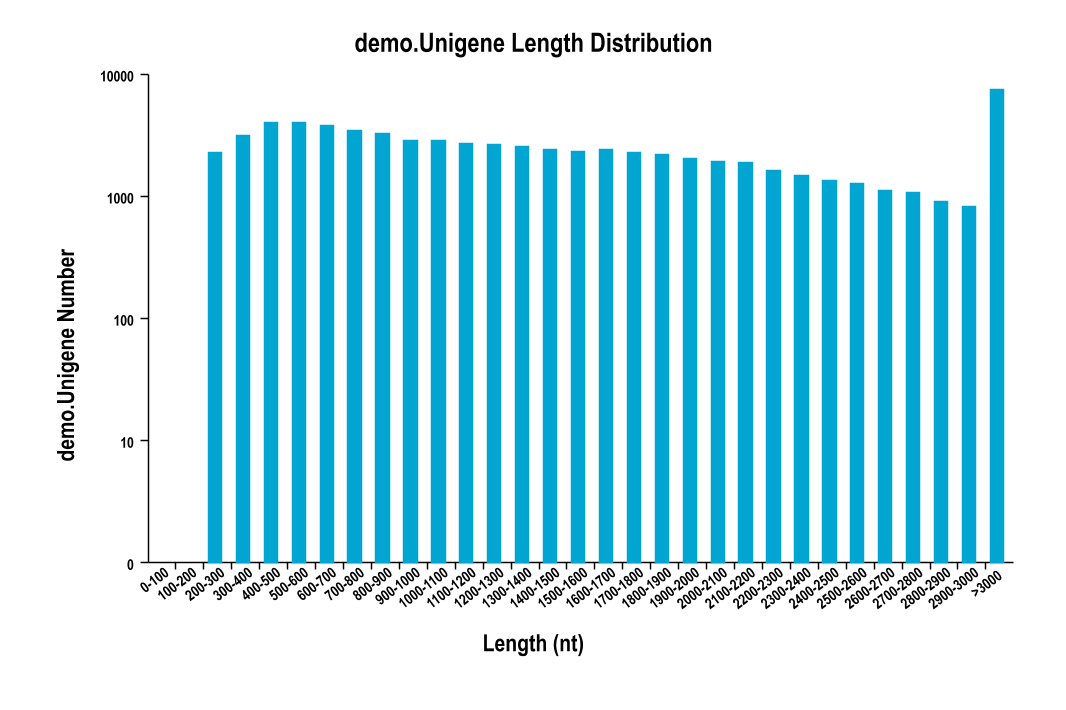

Supplement: Supplementary file 1 [file genes-14-00766-s001.zip › Figure S2.png]

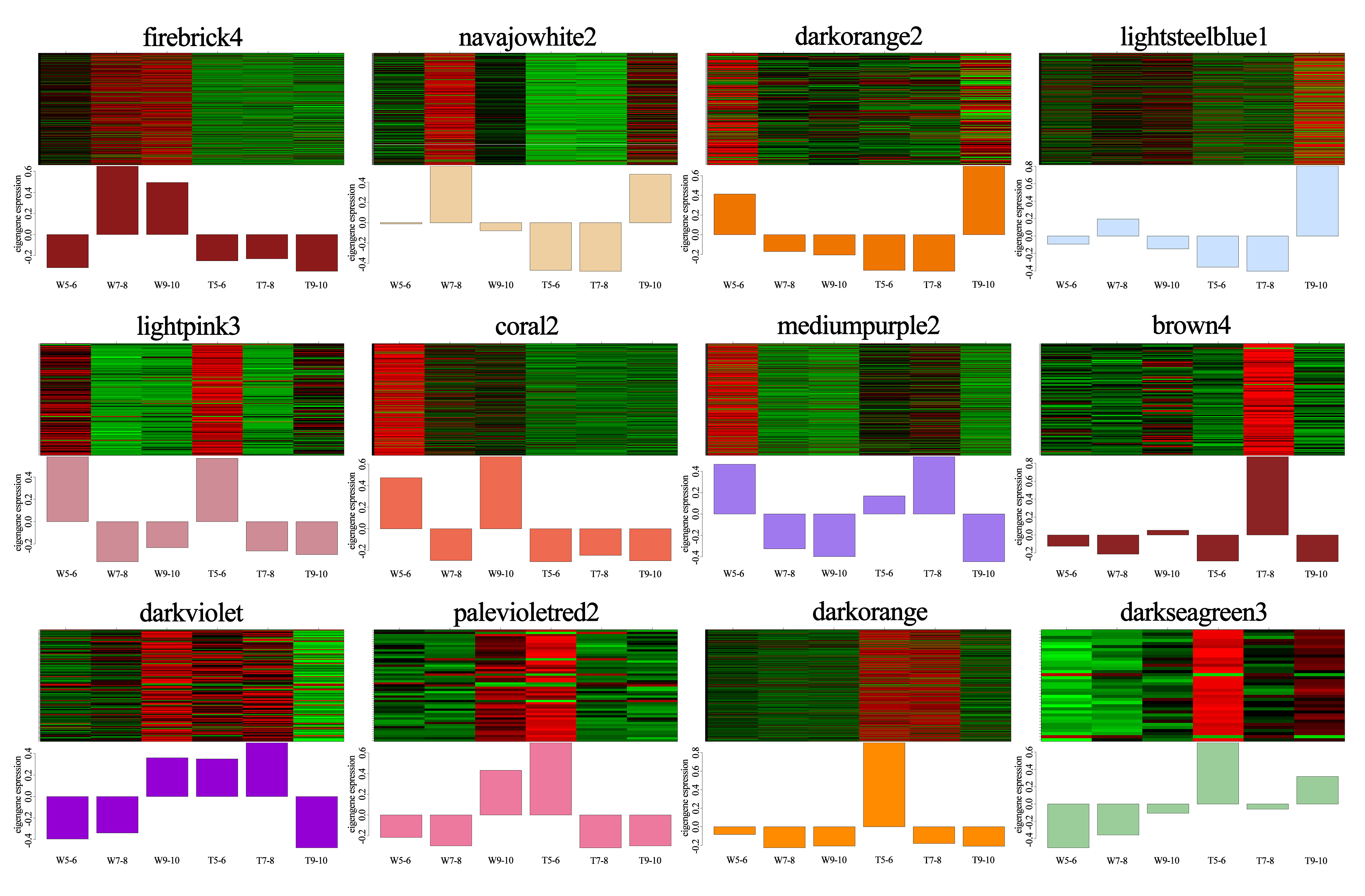

Supplement: Supplementary file 1 [file genes-14-00766-s001.zip › Figure S3.jpg]
